# The role of membrane acid/base transporters and carbonic anhydrases for cellular pH and metabolic processes

**DOI:** 10.3389/fnins.2014.00430

**Published:** 2015-01-05

**Authors:** Joachim W. Deitmer, Shefeeq M. Theparambil, Iván Ruminot, Holger M. Becker

**Affiliations:** ^1^General Zoology, FB Biology, University of KaiserslauternKaiserslautern, Germany; ^2^Zoology/Membrane Transport, FB Biology, University of KaiserslauternKaiserslautern, Germany

**Keywords:** pH, bicarbonate, lactate, transport metabolon, metabolism

## Acidosis and proton homeostasis in cells and tissues

Acidosis in the brain may severely impair a variety of functions, including synaptic transmission, metabolic energy supply, membrane transport and other processes (Ruusuvuori and Kaila, 2014).

Transport of acid–base equivalents across the cell membrane of neurons and glial cells also results in pH changes in the extracellular spaces. Cytosolic and extracellular buffer capacity and the activity of carbonic anhydrases contribute to shape pH changes, which can be elicited by neuronal activity, neurotransmitters and neuromodulators, metabolic processes, active cellular pH regulation, and secondary transporters carrying acid–base equivalents, and in turn these pH changes can affect neuronal functions (Deitmer and Rose, 1996; Chesler, 2003). The free H^+^ concentration in cells is in the nanomolar range, and the high buffer capacity of cells provides a reservoir of acid equivalents in the millimolar range. In other words, there is a pool of protons in rapid exchange between buffer sites and free solution, with 10^5^ or more protons being buffered for each proton in solution. At a blood pH of 7.4, and 7.2–7.3 in the extracellular spaces of brain tissue (Cragg et al., 1977; Ruusuvuori and Kaila, 2014), and with a negative membrane potential of between −50 and −90 mV in mammalian brain cells, H^+^ has to be continuously extruded to maintain a physiological cytosolic pH of 7.0–7.3. Nevertheless, pH changes may peak well outside this range, at least for short time periods, and may be considered as H^+^ signals, sometimes even with neurotransmitter function (Deitmer and Rose, 1996; Du et al., 2014). The net extrusion of acid from neurons and glial cells is accomplished by secondary active transport, wherein the efflux of H^+^ or the influx of HCO^−^_3_ is coupled to Na^+^ influx, utilizing energy stored in the transmembrane Na^+^ gradient. pH regulation in these cells involves a variety of membrane acid–base carriers, including sodium–hydrogen exchange, sodium–bicarbonate cotransport, and sodium-dependent and sodium-independent chloride–bicarbonate exchange. In addition, there are a number of acid/base-coupled carriers, which are linked to the transport of metabolites, such as lactate and amino acids. The lactate transport via monocarboxylate transporters (MCTs) has been suggested to play a major role for the supply of energy to neurons, and led to the “Astrocyte-to-Neuron Lactate Shuttle Hypothesis” (ANLSH; Pellerin and Magistretti, 1994).

## Lactate shuttle and acid/base transport metabolon

Lactate, pyruvate, and ketone bodies are transported into and out of cells via MCTs (*SLC*16), of which 14 isoforms have been described. The first four of these 14 isoforms (MCT1-4) have been shown to transport monocarboxylates together with H^+^ in a 1:1 stoichiometry. MCT1 is the ubiquitous isoform that is found in nearly all tissues, where it could either operate as a lactate importer or exporter, and has an intermediate *K_m_* value of 3–5 mM for L-lactate (Bröer et al., 1998). MCT2, the high-affinity carrier, is mainly found in neurons, and MCT4, the low-affinity, high-capacity carrier, has been reported for glial cells in the brain.

The lactate shuttle hypothesis suggests that lactate is produced and exported by glial cells, in particular astrocytes, under normoxic conditions, and taken up by neurons for further metabolization (Pellerin and Magistretti, 1994). The ANLSH infers that astrocytes help to supply energetic substrates for neurons to meet their energy requirements, especially during enhanced neuronal activity. There is substantial evidence, both *in vitro* and *in vivo*, that lactate indeed can substitute for glucose to maintain neuronal functions, such as e.g., synaptic transmission and memory formation (Schurr et al., 1988; Suzuki et al., 2011). During energy deprivation, the addition of monocarboxylates has been shown to restore synaptic function and to be neuroprotective *in vivo*, in acute rodent brain slices, isolated optic nerve and neuronal cultures (Izumi et al., 1997; Schurr et al., 1997; Cater et al., 2001; Wyss et al., 2011). The finding that glucose is preferentially taken up by astrocytes and at higher rates than by neighboring neurons (Barros et al., 2009; Jakoby et al., 2014), implying that some energetic substrate has to be passed on to neurons, as they are the main energy consumers, also supports the ANLSH. More recently, lactate production and supply to neuronal axons have been suggested also for oligodendrocytes in the mammalian central nervous system (Fünfschilling et al., 2012; Lee et al., 2012), indicating that astrocytes and oligodendrocytes form a metabolic network with neurons to maintain neuronal function.

A transport metabolon has been defined as a supramolecular complex of sequential metabolic enzymes and cellular structural elements in which metabolites are passed from one active site to another without complete equilibration with the bulk cellular fluids (Srere, 1985). First evidence for a transport metabolon, formed between carbonic anhydrase (CA) and an acid/base transporter was found for CAII and the Cl^−^/HCO^−^_3_ exchanger AE1 (Kifor et al., 1993; Vince and Reithmeier, 1998). Since then, various acid/base transporters have been reported to interact with different isoforms of carbonic anhydrase: For the electrogenic Na^+^/HCO^−^_3_ cotransporter, NBCe1, both functional (Becker and Deitmer, 2007; Schüler et al., 2011) and physical (Gross et al., 2002; Alvarez et al., 2003; Pushkin et al., 2004) interaction with different CA isoforms has been suggested. All of these interactions have in common that CA-mediated augmentation of transport activity requires the catalytic activity of the different CA isoforms.

An entirely different form of transport metabolon has first been detected, when expressing MCT1 and CAII in *Xenopus* oocytes (Becker et al., 2005). The presence of CAII indeed more than doubled the rate of lactate transport, and the CAII-induced augmentation of MCT activity persisted in the absence of CO_2_/HCO^−^_3_, and was insensitive to inhibition of CAII catalytic activity with EZA, and was still present with the catalytically inactive mutant CAII-V143Y (Becker et al., 2005, 2011; Becker and Deitmer, 2008), suggesting that the augmentation of MCT activity does not depend on the reversible conversion of CO_2_ and HCO^−^_3_/H^+^ by CAII. No interaction between CAII and rat MCT2 could be detected, when the enzyme was injected into oocytes co-expressing MCT2 together with its trafficking protein embigin (Klier et al., 2011). Cytosolic CAII was shown to bind to the C-terminal tail of MCT1, which presumably positions the enzyme close enough to the pore of the transporter for efficient H^+^ shuttling (Stridh et al., 2012). The binding of CAII to a glutamic acid cluster within the MCT C-terminal may also explain the isoform specificity of the interaction between MCTs and CAII, since rat MCT4, but not MCT2, possesses a similar cluster of three glutamate residues.

Augmentation of MCT activity by extracellular CAs has also been found in the brain: By inhibition of extracellular CA activity with benzolamide and an antiserum against CAIV, respectively, Svichar and Chesler (2003) could show a significant reduction in lactate-induced intracellular acidification in rat hippocampal pyramidal neurons and in cultured astrocytes.

## CA activity mediates between different forms of metabolic acidosis

Carbonic anhydrases play a vital role in acid/base kinetics and mediate between acid production by oxidative phosphorylation in form of CO_2_ and acid production by anaerobic glycolysis. When CO_2_ increases in the cell, e.g., due to oxidative phosphorylation in mitochondria, it can leave the cell by freely diffusing through the cell membrane, or it can be converted to H^+^ and HCO^−^_3_, with the rate of conversion depending on catalytic activity of cytosolic CA. Most cells express CAII, which is the fastest isoform, and either CAIV and/or CAXIV, which are fast extracellular isoforms in the brain. CAIV has recently been shown to display intracellular activity in addition, which would further contribute to high intracellular CA activity (Schneider et al., 2013). With this enzymatic equipment, neurons and glial cells can produce considerable amounts of H^+^, which can be extruded by either NHE or MCT. Extracellular CA activity can convert part of extracellular CO_2_ to H^+^ and HCO^−^_3_, the latter being substrate for NBC to be transported into and out of the cell. Thus, additional HCO^−^_3_ can be delivered to, or removed from, the cytosol, in particular in astrocytes, which can have a robust expression of NBC, which mediates a high bicarbonate sensitivity of the cells, to further compensate metabolically produced H^+^ (Theparambil et al., 2014).

Furthermore, both extra- and intracellular CA isoforms, as e.g., CAIV, can form transport metabolons with the bicarbonate- and proton-coupled carriers (see above). In mouse retina, CAXIV co-localized with anion exchanger isoform 3 (AE3) in Müller and horizontal cells, and physical and functional interaction between the CAXIV and AE3 was shown (Casey et al., 2009). Disruption of transport metabolon function, as suggested to occur after CAIV mutation, can interfere with photoreceptor maintenance and pH regulation in the retina (Yang et al., 2005; Alvarez et al., 2007). Whether other extracellular CA isoforms, which have been detected in brain tissue, also form functional metabolons with MCT and/or NBC, is still unknown. Interestingly, cytosolic CAI and CAIII, which are expressed by some cells, can enhance NBC activity in *Xenopus* oocytes (Schüler et al., 2011), but not MCT transport activity (Becker and Deitmer, 2008). In addition, by stabilizing the H^+^ gradient, NBC can support lactate transport via MCT, when expressed together in oocytes (Becker et al., 2004).

From these and other results, it can be concluded that brain cells, and quite possibly other cell types in other tissues, use a whole network of acid/base-coupled membrane carriers and different CA isoforms to regulate intracellular pH, which links acid/base status, H^+^ buffering, energy metabolism, and H^+^/HCO^−^_3_-coupled membrane transport. Thus, acid/base-coupled metabolite transport is coupled to pH regulation, and both are linked to CA activity and to non-catalytic functions of CA.

## Conclusions and perspectives

Regulation of metabolism in organisms is not only complex, but also involves a large number of enzymes and membrane transporters, which may form networks to enhance their efficacy. Lactate, as a metabolic intermediate from glucose or glycogen breakdown, appears to play a major role as energetic substrate shuttled between cells and tissues, both under hypoxic and normoxic conditions. The membrane transport of lactate via monocarboxylate transporter occurs in cotransport with H^+^, which is a substrate, a signal and a modulator of other metabolic processes. Lactate transporter form a “transport metabolon” with carbonic anhydrases, which not only provide a rapid equilibrium between CO_2_, HCO^−^_3_, and H^+^, but in addition enhance lactate transport by a non-enzymatic interaction, which requires physical binding as found in frog oocytes as expression system for the proteins involved. Carbonic anhydrases mediate between different states of metabolic acidosis, induced by glycolysis and oxidative phosphorylation, and play a relay function in coupling pH regulation and metabolism.

### Conflict of interest statement

The authors declare that the research was conducted in the absence of any commercial or financial relationships that could be construed as a potential conflict of interest.
